# Augmentative Biological Control Using Parasitoids for Fruit Fly Management in Brazil

**DOI:** 10.3390/insects4010055

**Published:** 2012-12-21

**Authors:** Flávio R. M. Garcia, Marcelo P. Ricalde

**Affiliations:** Insect Ecology Lab, Department of Ecology, Zoology and Genetics, Institute of Biology, Federal University of Pelotas, P.O. Box 354, Pelotas, RS CEP 96010-900, Brazil; E-Mail: flaviormg@hotmail.com

**Keywords:** fruit flies, parasitoid, Diptera, Tephritidae, Braconidae

## Abstract

The history of classical biological control of fruit flies in Brazil includes two reported attempts in the past 70 years. The first occurred in 1937 when an African species of parasitoid larvae (*Tetrastichus giffardianus*) was introduced to control the Mediterranean fruit fly, *Ceratitis capitata* and other tephritids. The second occurred in September 1994 when the exotic parasitoid *Diachasmimorpha longicaudata*, originally from Gainesville, Florida, was introduced by a Brazilian agricultural corporation (EMBRAPA) to evaluate the parasitoid’s potential for the biological control of *Anastrepha* spp. and *Ceratitis capitata*. Although there are numerous native Brazilian fruit fly parasitoids, mass rearing of these native species is difficult. Thus, *D. longicaudata* was chosen due to its specificity for the family Tephritidae and its ease of laboratory rearing. In this paper we review the literature on Brazilian fruit fly biological control and suggest that those tactics can be used on a large scale, together creating a biological barrier to the introduction of new fruit fly populations, reducing the source of outbreaks and the risk of species spread, while decreasing the use of insecticides on fruit destined for domestic and foreign markets.

## 1. Introduction

Approximately 70 species of fruit flies (Diptera: Tephritidae) are considered important agricultural pests [1]. These tephritids are mainly found in fruit crops including oranges, mangoes, apples and peaches, and fall into five genera: *Anastrepha *Schiner, *Bactrocera *Macquart, *Ceratitis *MacLeay, *Dacus *Fabricius and *Rhagoletis *Loew [1]. In Brazil, fruit fly larvae cause major production losses and limit fresh fruit exports mainly due to quarantine measures imposed by importing countries [2]. Economic losses due to fruit flies exceed two billion dollars worldwide [3]. In Brazil, more than 50% of the economic losses have been recorded from the host plant families, Anacardiaceae, Myrtaceae, Passifloraceae and Sapotaceae [4]. Across South America, *Anastrepha fraterculus* Wiedemann (Diptera: Tephritidae) is of great economic importance across a variety of different environments. In Argentina, Uruguay and states of the southern and southeastern regions of Brazil (from Rio Grande do Sul to the south-central areas of Minas Gerais and Espirito Santo), this species is a serious pest, requiring the adoption of control measures to prevent economic losses [2].

Many natural enemies that are responsible for regulating populations, either naturally or by means of augmentative releases, have been identified [5]. The majority of fruit fly natural enemies in the Neotropical region belong to the families Braconidae (Opiinae), and Figitidae [6]. The most important biological control agents in Brazil belong to the genera *Opius *Wesmael, *Utetes *Foerster, *Doryctobracon *Szépligeti, *Aganaspis *Brèthes, *Biosteres *Ashmead and *Diachasmimorpha *Ashmead [5]. Augmentative biological control is a strategy whereby a natural enemy present in crops is augmented by individuals reared in the laboratory and released into the targeted area. The objective of an augmentative release is to cause rapid mortality in the host population [7]. Natural enemies are mass reared in specialized laboratories; therefore, this strategy relies on the ability to effectively mass produce natural enemies [7]. The timing of release is based on the biology of the target pest and the release is synchronized with periods when the pest is at the most susceptible stage [8]. 

Several countries produce and release parasitoids on a large scale, seeking to control and reduce populations to economically acceptable levels [9]. The first experiments with production-scale releases of braconids to suppress fruit flies were conducted in Hawaii [10]. Those promising results encouraged similar projects in Costa Rica, Mexico, Guatemala, United States (Florida) and Brazil [10,11]. The exotic parasitoid *Diachasmimorpha longicaudata* (Ashmead) (Hymenoptera: Braconidae) which has been used for biological control of *Anastrepha* populations, was originally collected from the Malaysia-Philippine region and is today considered one of the most important larval parasitoid biological control agents. This importance is based on its ease of rearing in the laboratory, the ability of *D. longicaudata* to quickly adapt to different environments, and its specificity of parasitism on tephritids and high capacity for parasitism on fruit flies [12,13].

Although *D. longicaudata *has multiple fruit fly hosts and is easily reared in the laboratory, efforts to repeat the success of previous biological control campaigns have failed in the State of Rio Grande do Sul, where the climate is believed to have been the main limiting factor [13]. The potential of native parasitoids for the biological control of fruit flies is therefore being explored in this region [9]. A number of candidate species have been identified, including *Opius* sp. (Hymenoptera: Braconidae), *Doryctobracon brasiliensis* (Szépligeti) (Hymenoptera: Braconidae), *Doryctobracon areolatus* (Szépligeti) (Braconidae) and *Aganaspis pelleranoi *(Brèthes) (Hymenoptera: Figitidae), which in many situations can be found in orchards, together parasitizing up to 40% of flies [9]. Of these species, *D. areolatus *is the most abundant and frequent in Brazil [9].

The aim of this review is to discuss the history and current state of augmentative biological control of fruit flies in Brazil. 

## 2. Native Parasitoids

Brazil is home to many species of native parasitoids (Table 1), including the efficient parasitoid *D. areolatus *which is widely distributed across the country. However, to date, the mass rearing of this and other native parasitoids has not been successful. *Doryctobracon areolatus* is only attracted to fruit fly larvae when they are associated with the fruit, thus increasing production costs and preventing their use in biological control in Brazil [14].

Nine native parasitoid species from the family Braconidae have been identified in several states of Brazil and other South American countries, but the most promising species for applicability in biological control programs include *D. areolatus*, *Opius bellus* Gahan and *Utetes anastrephae *(Viereck) (Braconidae), due mainly to their abundance in many regions [10]. In Bahia, five species of the fruit fly genera *Anastrepha* were reported: *A. fraterculus *(Wiedemann), *Anastrepha sororcula *Zucchi, *Anastrepha obliqua *(Macquart), *Anastrepha*. *serpentina *(Wiedemann) and *Anastrepha bahiensis *Lima. In addition, three Braconidae species were found: *D. areolatus*, *Utetes anastrephae *(Viereck) and *Asobara anastrephae *(Muesebeck) (Braconidae), with a parasitism index of 0.63% in Surinam cherry (*Eugenia uniflora*) and 8.97% in letterhout (*Helicostylis tomentosa*) [15]. In the Central Amazon region, *Anastrepha pulchra *Stone was collected from *Mouriri collocarpa *Ducke (Melastomataceae), in association with the parasitoid *D. areolatus*; *Anastrepha atrigona *Hendel in *Strychnos jobertiana *Baillon (Loganiaceae) and *Pouteria durlandii *(Standley) Baehni (Sapotaceae), in association with the parasitoids *Opius bellus*, *Opius *sp. (Braconidae), *Aganaspis pelleranoi *(Brèthes) (Figitidae) and *Anastrepha coronilli *Carrejo and González, in association with the parasitoid *Aganaspis nordlanderi *Wharton (Figitidae) [16]. In Santa Catarina, the main fruit fly parasitoids belong to three families: Braconidae, Diapriidae and Figitidae. Nora *et al. *[17] reported that *D. areolatus* (Szépligeti), *D. brasiliensis *Szépligeti, *Opius bellus *Gahan, 1930 and *Opius. tomoplagiae *(Lima), with *D. areolatus *were the most abundant. In that study, other parasitoids from the families Diapriidae, Eulophidae, Pteromalidae and Figitidae were obtained but not identified. Guimarães *et al*. [18] identified two Figitidae species, not previously recorded from this region, *A. pelleranoi* and *Odontosema anastrephae *Borgmeier (Figitidae)*. *Leonel Jr. *et al*. [19] and Canal and Zucchi [6] have reported the occurrence of the braconids *Microcrasis lonchaea *(Lima) (Braconidae) and *Utetes anastrephae* (Braconidae)*.* More recently, Garcia and Corseuil [20] recorded *Trichopria anastrephae *Lima (Diapriidae) and *Lopeucoila anastrephae *(Rhower*)* (Figitidae) in the region.

Recent studies of the biology of one of the most promising parasitoids, *D. areolatus*, demonstrated average parasitism rates of *A. fraterculus* of 54% could be achieved, with a range of 41.6% to 68.6%. The development of the parasitoid from larva to adult took 25 days, with an average longevity of 16 days for males and 10 days for females and a sex ratio of 0.62, demonstrating the species’ capacity to parasitize *A. fraterculus* [21].

**Table 1:** List of parasitoids and their fruit fly hosts in Brazil.

**Species**	**Family of parasitoid**	**Host/s**	**State** [Reference]
*Aganaspis nordlanderi *Wharton	Figitidae	*A. bahiensis*, *A. coronilli*, *A. strita Neosilba *sp.	**AM** [16,18], **SP** [22]
*Aganaspis pelleranoi *(Bréthes)	Figitidae	*A. amita*, *A. bahiensis*, *A. fraterculus*, *A. atrigona*, *A. serpentina*, *Ceratitis capitata*, *Neosilba *sp., *N. pendula*, *N. perezi*, *C. capitata*	**AM** [16,18], **BA** [23], **GO** [24], **PR** [25], **RJ** [18], **RS** [26,27,28], **SC** [18,20,29], **SP** [29,30]
*Asobara anastrephae *(Muessebeck)	Braconidae	*Anastrepha *sp., *A. Bahiensis*, *A. obliqua*, *A. Zenildae*	**AM** [31,32], **BA** [15,33], **GO** [19,24], **MS** [34,35], **RN** [36], **SP** [19]
*Asobara *sp.	Braconidae	*-*	**SP** [37]
*Coptera haywardi* Loiácono	Diapriidae	*A. fraterculus*, *A. sororcula*	**RJ** [38]
*Dicerataspis flavipes* (Kieffer)	Figitidae	*A. amita*	**PA** [39], **SP** [18]
*Doryctobracon areolatus *(Szépligeti)	Braconidae	*A. amita*, *A. bahiensis*, *A. bistrigata*, *A. fraterculus*, *A. leptozona*, *A. obliqua*, *A. pelleranoi*, *A. pulchra*, *A. pseudoparallela*, *A. serpentina*, *A. sororcula*, *A. striata*, *A. zenildae*, *C. capitata*, *R. pastranai*, *Neosilba *sp.	**AM** [16,29,31], **BA** [15,19,40], **ES** [19], **GO** [19,24], **MA** [41], **MS** [34,42], **MG** [6,43], **PA** [44], **PR**, **PI** [45], [9,25], **RN** [36], **RS** [19,26,27,28], **RJ** [19,46], **SC** [17,19,20], **SP** [19,37,47,48,49,50]
*Doryctobracon brasiliensis *(Szépligeti)	Braconidae	*A. amita*, *A. fraterculus*, *A. serpentina*, *A. pelleranoi*, *A. sororcula*, *O. anastrephae*, *R. pastranai*	**AM** [29], **PR** [25], **RS** [19,26], **RJ** [19,46], **SC** [17,19,20], **SP** [19,47,48,50]
*Doryctobracon fluminensis *Lima	Braconidae	*A. fraterculus*, *A. paralela*, *A. pseudoparallela*, *A. pickeli*, *A. montei*, *Hexachaeta eximia*	**BA** [51], **MG** [6,43], **MS** [34,35], **RJ** [29,52]
*Doryctobracon *sp.	Braconidae	*-*	**GO** [24]
*Idiasta delicate *		*Anastrepha *sp.	**AM** [32]
*Lopheucoila anastrephae *(Rhower)		*A. amita*, *A. Pseudoparallela*, *Neosilba *sp., *Lonchaea* sp.	**MS** [22], **SC** [20], **SP** [29]
*Lopheucoila truncicola *Weld*.*	Figitidae	*-*	**RJ** [53]
*Microcrasis lonchaeae* (Lima)	Braconidae	*Rhagoletotrypeta pastranai*, *Neosilba pendula*	**SC** [17], **SP** [29]
Opius bellus Gahan	Braconidae	A. fraterculus, A. pickeli, A. montei, A. obliqua, A. serpentina, A. atrigona, C. capitata, Rhagoletis ferrugínea, T. anastrephae, R. pastranai	**AM** [16,31], **GO** [24], **MS** [34,42], **PA** [44], **RS** [19,26,27,28,29], **RJ** [19,46], **SC** [17,19,20], **SP** [19,29,43,47,49]
Opius bucki Lima	Braconidae	Tomoplagia rudolphi	**MG** [29], **RS** [52]
Opius itatiayensis Lima	Braconidae	Tomoplagia sp.	**RJ** [52]
Opius tomoplagiae Lima	Braconidae	Tomoplagia rudolphi	**MG** [52], **PR** [29], **RJ** [52], **SP** [29]
Opius sp.	Braconidae	Anastrepha sp, A. distincta, A. atrigona, A. leptozona, A. obliqua	**AM** [16,31], **BA** [3], **GO** [24], **MS** [34], **PA** [29,44], **PI** [45], **RJ** [19,29], **SC** [17,19,20], **SP** [19]
Odontosema albinerve Kieffer	Figitidae	-	**PA** [39]
Odontosema anastrephae Borgmeier	Figitidae	A. fraterculus	**A M** [18], **MS** [18], **RS** [26], **SC** [18,20], **SP** [54]
Trichopria anastrephae Lima	Diapriidae	A. fraterculus, A. serpentina	**GO** [55], **MG** [56], **RS** [27], **RJ** [29,57,58], **SC** [20]
Tropideucoila angrensis Borgmeier	Figitidae	-	**RJ** [57]
Tropideucoila rufipes Ashmead	Figitidae	-	**MT** [59]
Tropideucoila weldi Lima	Figitidae	Neosilba pendula	**RJ** [54]
Trybliographa sp.	Figitidae	Anastrepha spp., Neosilba spp.	**SP** [18]
Utetes anastrephae (Viereck)	Braconidae	A. amita, A. fraterculus, A. obliqua, A. sororcula, A. manihoti, R. pastranai, Neosilba sp.	**AM** [31], **BA** [15,19,40,60], **MS** [34], **MG** [6] **PA** [44], **PR** [25], **RS** [19,26], **RJ** [19,46], **SC** [17,19,20], **SP** [43,47,48,60]

* AM = Amazonas, AP = Amapá, BA = Bahia, ES = Espírito Santo, GO = Goiás, MA = Maranhão, MG = Minas Gerais, MS = Mato Grosso do Sul, MT = Mato Grosso, PA = Pará, PI = Piauí, PR = Paraná, RN = Rio Grande do Norte, RJ = Rio de Janeiro, SC = Santa Catarina, SP = São Paulo.

## 3. Introduced Parasitoids

### 3.1. Tetrastichus giffardianus

Two classical biological control programs for the control of fruit flies have been reported in Brazil, and which preceded any form of augmentative release. The first occurred in 1937 when the African larval parasitoid *Tetrastichus giffardianus* Silvestri (Hymenoptera: Eulophidae) was introduced to control *Ceratitis capitata* Wiedemann and other tephritids. The second occurred in 1994 when the Brazilian agricultural corporation Embrapa Cassava and Tropical Fruits (“Embrapa Mandioca e Fruticultura Tropical”) introduced the exotic parasitoid *Diachasmimorpha longicaudata* (Ashmead) to Brazil from Gainesville, Florida, to evaluate the parasitoid’s potential for the biological control of fruit flies of the genus *Anastrepha* and *C. capitata* [11,61,62,63].

The use of *T. giffardianus *for the augmentative biological control of fruit flies has advantages and disadvantages. Advantages include the gregarious nature of this species, the high proportion of females among the offspring, and females which penetrate the fruit to lay eggs in the larvae, thus directly attacking the larvae that often escape from Opiinae parasitism, of which the larger fruit of the latter may be an issue, due to difficulties of the parasitoids ovipositor reaching deeply entrenched larvae [64]. Additionally, during the mass-rearing process, the time lag between the emergence of the flies and that of the parasitoids eliminates the problem of releasing these flies into the environment [63]. Disadvantages that may possibly limit the applicability of this parasitoid include its relatively low rate of parasitism and short life span when released. However, the low rate of parasitism would be offset by the high gross reproductive rate because more than one parasitoid is produced by the host. This parasitoid was introduced in the state of São Paulo, and 60 years later, it is no longer found in the southeast region. However, it persists in the states of Bahia, Rio Grande do Norte and Ceará, likely due to the warmer climate [63].

### 3.2. Diachasmimorpha longicaudata

Four years after *D. longicaudata*’s introduction into Bahia, Matrangolo *et al. *[65] conducted field surveys to evaluate the parasitoid’s establishment and association with several species of fruiting trees including: the common guava, *Psidium guajava* L. (Mirtaceae); Surinam cherry, *Eugenia uniflora *L. (Mirtaceae); starfruit, *Averrhoa carambola *L. (Oxalidacea); and mango, *Magnifica indica *Linnaeus (Anacardiaceae). Subsequently, the exotic *D. longicaudata *was mass reared in the laboratory and released weekly on all of the aforementioned fruiting trees but at low levels of approximately 1,000 parasitoids. Surveys prior to these releases in a multi-species orchard revealed the presence of a number of native hymenopterans, including *D. areolatus*, *Utetes anastrephae*, *Opius *spp., *Asobara anastrephae *(Muesebeck) and *Aganaspis pelleranoi*. Surveys conducted after the release of *D. longicaudata *showed that these native species persisted and that although interspecific competition for oviposition sites existed, no loss of biodiversity was observed [66]. *Diachasmimorpha longicaudata* was detected 17 months post release from starfruit, guava and other environments, such as a hybrid species of umbu‑caja, but was not recorded during the surveys conducted in 2004 and 2005 [66]. The ability of *D. longicaudata* to adapt to semi-arid (São Francisco water basin, State of Bahia, Brazil) and Amazonian (Oiapoque, State of Amapa, Brazil) ecosystems demonstrates this species’ capacity to parasitize hosts and complete its life cycle in a variety of different environments. Although the Amazon in particular presents complex ecosystems and interspecific competition with native species, this parasitoid was able to readily adapt [67].

Approximately 34,000 pairs of *D. longicaudata* were released on fifteen occasions over seven weeks in commercial guava orchards in the semi-arid northern region of Minas Gerais. Seven days *post* release, a survey recovered a total of 37 specimens of *D. longicaudata*, indicating that the species completed its life cycle in the local conditions and has the capacity to establish in the region [64,68,69].

In Botucatu, State of São Paulo, *D. longicaudata* were released in areas of riparian forest to suppress the density of *Anastrepha fraterculus* impacting nearby citrus orchards [70]. The release was timed to occur between insecticide applications, to minimize any effects of spray drift on released parasitoids. A total of 14 releases occurred between April and October, and a total of 560,000 parasitoids were released, which resulted in a 30% reduction in *A. fraterculus* infestations and a consequent reduction in the application of insecticides [70]. 

## 4. Mass Rearing and Release Requirements for Parasitoids in Brazil

Although Brazil has a diverse number of native fruit fly parasitoid species, difficulties have been found in the mass rearing of these species. Such difficulties led to the introduction of *D. longicaudata*. This parasitoid has been used because of its host specificity for the family Tephritidae and its ease of laboratory rearing. The parasitoid *D. longicaudata* originates from Indo-Australia where the natural hosts are flies of the genus *Bactrocera *[71].

When searching for a host, *D. longicaudata* is attracted to volatile substances released by the fruit and the vibrations produced by the fly larvae during feeding, mainly in fallen fruits rotting on the ground [72,73]. The average life cycle for *D. longicaudata* reared in the laboratory at a temperature of 22–26 °C, and 14-hour photoperiod on *C. capitata *larvae ranges from 14 to 16 days [73]. The pre-oviposition period is two days, and the oviposition period is 28–30 days. In mass-rearing facilities in the State of São Paulo, an average parasitism rate of 46.1% is achieved, with a ratio of three females to one male [73].

In Brazil, in studies with rearing cages placed under natural (outdoor) conditions, *D. longicaudata* was more efficient compared to other parasitoids and, in some cases, reached parasitism levels of 91.42%. This efficiency is true when reared on both *C. capitata* and *Anastrepha* sp. [14,67,74]. In the field, a significant reduction (40.0%–95.0%) was observed in trapped *A. suspensa* flies when the parasitoid was released at inundation [68,75,76,77,78,79].

Although *D. longicaudata* attacks both *C. capitata*, and *A. fraterculus*, when these species are present together in the same environment, the parasitoid demonstrates a preference for *A. fraterculus* [68].

**Figure 1:**
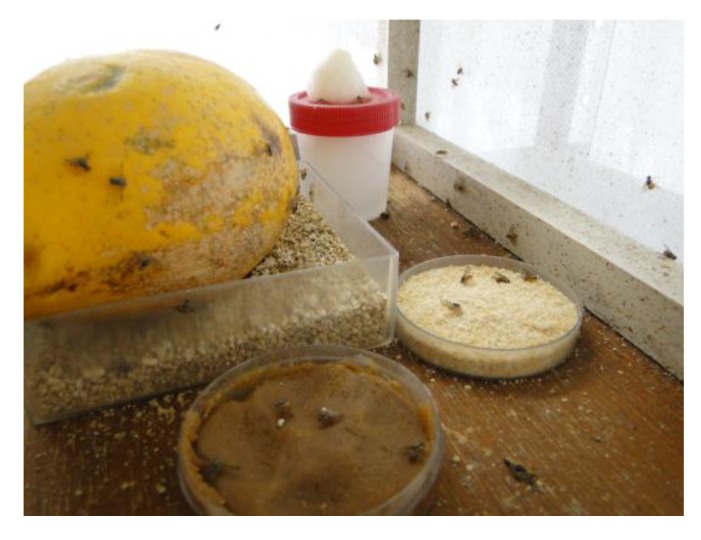
*C. capitata* in a rearing cage, water; artificial fruit; adult food; papaya.

In Brazil, * Diachasmimorpha longicaudata* is mass-reared in cages containing water, food (sugar and soy protein) and a means for oviposition (papaya fruit and artificial plastic fruit, although the flies will also oviposit through the cage screen) (Figure 1) [61]. The eggs are collected with a paintbrush, and the larvae are subsequently reared on an artificial diet. When the larvae reach third instar, they are exposed to adult parasitoids in a nursery container, simulating an infested fruit to facilitate access to the larvae. Adult parasitoids are fed an artificial diet (120 mL warm water, 0.8 g agar, 0.05 g ascorbic acid, 0.005 g Nipagin and 120 mL honey) and water. After 24 hours, the larvae are placed in containers containing vermiculite for pupation and adult emergence [61]. The parasitoid offspring are removed from the emergent cages with the aid of a vacuum adapted to provide weak suction [61]. 

Rearing of *D. areolatus* in Brazil is done in cages of varying sizes and always uses *A. fraterculus* as the host. The diet for this parasitoid is composed of honey, distilled water, agar, ascorbic acid, and sodium benzoate or honey and water. Mass production of this parasitoid has been unsuccessful because it is a species with a high risk of death due to behavioral attributes and genetic degeneration in the colonization process [14,21,80]. Additionally, the parasitoid *T. giffardianus* is a potential candidate for biological control in Brazil, but there is a lack of information about diet, mass-rearing methods and economics. On the other hand, *D. longicaudata* is easily reared and is the species most often utilized in Brazil. This species is primarily reared in larval *C. capitata* because they possess sterile lines, and the adults also utilize a diet based on honey, distilled water, agar, ascorbic acid and sodium benzoate (Table 2) [14,69,81,82]. 

**Table 2:** Comparison between the rearing techniques of *Doryctobracon areolatus *and *Diachasmimorpha longicaudata* in Brazil.

Parasitoids	*Doryctobracon areolatus*		*Diachasmimorpha longicaudata*		
Authors	Costa *et al.* [80]	Nunes *et al.* [21]	Walder *et al.* [81]	Alvarenga *et al.* [69]	Costa *et al.* [82]
Host	*A. fraterculus*	*A. fraterculus*	*C. capitata*	*C. capitata*	*C. capitata*
Cages (cm)	30 × 50 × 30	11 × 12 × 19	50 × 50 × 30	15 × 12	50 × 50 × 30
Diet	Honey, distilled water	Honey	Honey, distilled water, agar, ascorbic acid and sodium benzoate	Honey, distilled water	Honey, distilled water, agar, ascorbic acid and Nipagin
Temperature (°C)	25 ± 2	25 ± 2	26 ± 2	26 ± 2	26 ± 2
Humidity (%)	70 ± 10	70 ± 10	75 ± 5	65 ± 10	70 ± 10
Photophase (h)	4	12	14	14	14
Sex ratio	0.62	0.62 ± 0.09	———	———	0.57
mean longevity (days)	————	16.36 ± 3.62 (♂) and 10.24 ± 1.71 (♀)	———	————	————
mean parasitism rate (%)	————	53.50 ± 8.93	46.1%	————	————

In Brazil, there are few publications on biological parameters of parasitoids of fruit flies reared in the laboratory, which makes it difficult to determine the best technique. However, it is known that the mass-rearing technique used by the Center for Nuclear Energy in Agriculture, University of São Paulo (CENA/USP) produced more than 150 million specimens of *D. longicaudata* in Brazil from January 1995 to October 2001 [81].

## 5. Bio-Fabrication

Today, the biological control of fruit flies in programs across Brazil is largely based on the augmentative release of introduced parasitoids to achieve effective suppression of pest fly populations on an area-wide basis [62]. Consequently, for these programs to be successful, a large number of agents must be available. In Brazil, bio-factories mass produce and commercially provide a range of biological control agents. The bio-factory Moscamed Brazil was created in Juazeiro, State of Bahia, in 2005, with the main objective of producing sterile insects for the release and eradication of *C. capitata. *This factory has a production capacity of 100 million sterile males per week. In addition, the factory also produces 10 million *D. longicaudata*/week for the biological control of *Anastrepha* spp. and *C. capitata* [14]. The factory was made possible through investments by federal and state governments and international partnerships (International Atomic Energy Agency––IAEA).

## 6. Conclusions

In Brazil, the most effective parasitoid for use in augmentative biological control programs for *Anastrepha* spp. and *C. capitata* is *D. longicaudata. *This species is preferred mainly due to its ease of rearing and high parasitism levels. Successful control programs with this parasitoid require repeated releases until the parasitoid becomes fully established in the area [62].

Worldwide, *D. longicaudata* is an important parasitoid of fruit flies for several reasons including its ease of rearing, intensive foraging and high activity of resource exploitation by females. However, several studies have shown low indices of parasitism and recovery of specimens of *D. longicaudata* released in Brazil. Evidence from these studies suggests that the removal of fruit from the field during sampling may have reduced the susceptibility period of larvae to the parasitoid, therefore generating an underestimation of the parasitism index. Moreover, *D. longicaudata* also demonstrates a low tolerance to cold, inferred from its decreased effectiveness during the winter in southern Brazilian states, despite continued effectiveness in warmer regions of the country [63].

Studies that seek alternatives to using exotic introduced parasitoids in Brazil are greatly lacking, with more technically and economically feasible efforts required for the development of research aimed at the mass rearing of native parasitoids, particularly *D. areolatus*. Another parasitoid that requires research attention for its potential against a range of flies in Brazil is *Fopius arisanus *(Sonan). This parasitoid develops in the egg and early larval instar of fruit flies, and emerges from the pupae [83] and has surpassed *D. longicaudata* in all competition studies [84,85,86].

The successful use of parasitoids for the suppression of fruit fly populations depends on studies that evaluate the behavior of these insects and the use of such methods in conjunction with other area-wide control techniques on an integrated pest management basis (IPM). In Brazil, biological control and the sterile insect technique (SIT) are expected to be used across large areas, suppressing fly populations, reducing the source of infestations and the risk of spread of pest species, reducing the use of insecticides in fruit destined for domestic and foreign markets, and ultimately creating a biological barrier to fruit fly incursions in major horticultural regions.

The augmentative biological control of fruit flies is now a reality in Brazil, along with other biological control techniques, including SIT, which, when used in combination, is believed capable of increasing the overall efficiency and effectiveness of control of pest fruit flies in Brazil [87].
